# Head Nurse Leadership: Facilitators and Barriers to Adherence to Infection Prevention and Control Programs—A Qualitative Study Protocol †

**DOI:** 10.3390/nursrep14030138

**Published:** 2024-07-26

**Authors:** Eva Cappelli, Jacopo Fiorini, Francesco Zaghini, Federica Canzan, Alessandro Sili

**Affiliations:** 1 Department of Diagnostic and Public Health, University of Verona, Verona 37129, Italy; federica.canzan@univr.it; 2 Department of Biomedicine and Prevention, University of Rome Tor Vergata, Rome 00133, Italy; francesco.zaghini@uniroma2.eu; 3 Nursing Department Tor Vergata University of Rome Tor Vergata, Rome 00133, Italy; fiorini.jcp@gmail.com (J.F.); alessandro.sili@ptvonline.it (A.S.)

**Keywords:** cross infection, leadership, drug resistance multiple, nurses, qualitative research

## Abstract

Background: The effective management of Healthcare-Associated Infections (HAIs) relies on the implementation of good practice across the entire multidisciplinary team. The organizational context and the role of head nurses influence the team’s performance and behavior. Understanding how decision-making processes influence healthcare professionals’ behavior in the management of HAIs could help identify alternative interventions for reducing the risk of infection in healthcare organizations. This study aims to explore how the behaviors promoted and actions implemented by the head nurse can influence healthcare professionals’ adherence to Infection Prevention and Control (IPC) programs. Methods: A multi-center qualitative study will be conducted using a Grounded Theory approach. Observations will be conducted, followed by individual interviews and/or focus groups. A constructive and representative sample of healthcare professionals who care directly for patients will be enrolled in the study. The COnsolidated criteria for REporting Qualitative research (COREQ) checklist will be followed to ensure the quality of this study protocol. A multistep inductive process will be used to analyze the data. Conclusions: The study results will provide an understanding of how nurses perceive the influence of leadership and how they modify their behaviors and activities toward patients according to IPC programs. The study will identify barriers and facilitators to IPC compliance and suggest strategies to minimize negative patient outcomes, such as the development of an HAI.

## 1. Introduction

In recent years, increased attention has been focused on infections acquired by patients in healthcare facilities within 48 h of hospitalization [1], particularly those caused by multidrug-resistant microorganisms (MDROs), such as carbapenemase-resistant *Escherichia coli*, third-generation cephalosporin and carbapenemase-resistant *Klebsiella pneumoniae*, vancomycin-resistant *Enterococcus faecium*, carbapenemase-resistant *Acinetobacter*, and *Pseudomonas aeruginosa*. Each year, over 670,000 infections are caused by MDROs, resulting in 33,000 deaths due to the lack of effective treatment [2]. The spread of these infections is influenced by various factors, and infection development may be related to intrinsic patient conditions [3] and healthcare professionals’ adherence to Infection Prevention and Control (IPC) and antimicrobial stewardship programs [4,5]. In addition to these factors, it is possible to include others related to the organization of the hospital [6,7], the type of leadership used by managers and head nurses [8], the quality and quantity of resources available [9], and, most importantly, the culture of patient safety present in the hospital [10]. The head nurse, also known as nurse managers or charge nurses, are registered nurses responsible for the management and supervision of the nursing staff within a healthcare facility, department, or unit. They also guide, support, and motivate the entire clinical care team to work towards the achievement of objectives, ensuring high care quality [11]. Given this, the head nurse is also responsible for the prevention and control of Healthcare-Associated Infections, given his/her role, responsibilities, and skills required by the hospital. This can be achieved by promoting adherence to good healthcare practices among the team of healthcare professionals they manage, with the support of infection risk specialists [12]. The head nurse also serves as the link between the instructions of the organization’s management and the evidence-based practice activities of the healthcare professionals [13].

Within healthcare organizations, the head nurse manages and leads a team of individuals with diverse backgrounds and competencies. As a middle manager, their role is to improve the well-being of nurses, achieve high levels of performance, and ultimately improve the quality of care for patients [13,14,15].

According to the World Health Organization [2], promoting, supporting, and incentivizing educational pathways that prepare healthcare professionals to become hospital head nurses is critical. These pathways prepare healthcare professionals to provide effective leadership within their care settings and teams. Success can be achieved through the implementation of specialized skills and competencies such as systematic thinking, communication, negotiation, strategy, and analysis, as well as the development of a culture of learning [16].

These skills and competencies being used in clinical practice guarantee the following: (a) The acquisition of an effective and transparent method of communication capable of conveying concepts derived from evidence-based nursing practices [17]. (b) The overcoming of stress factors present within the team [18], mainly due to staff disagreements [19], which inevitably lead to burnout, job dissatisfaction [20], and the intention to leave [12]. (c) The identification and implementation of multimodal strategies to address emerging challenges such as HAIs caused by multidrug-resistant pathogens (MDROs), which are a serious concern worldwide [21,22,23].

### Theoretical Framework

Studies have shown that authentic and transformational leadership styles are positively related to HAIs [24,25], including device-associated infections like Catheter-Associated Urinary Tract Infections (CAUTIs) and Central Line-Associated Bloodstream Infections (CLABSIs) [26,27]. These findings may have been achieved because the head nurses encouraged their nurses to follow the Infection Prevention and Control (IPC) programs available within their hospital [22] to seek evidence-based solutions suitable for their work context; to use problem-solving and decision-making skills in complex situations [10,24] to share and collaborate clearly and transparently with the multidisciplinary team, breaking all institutional hierarchies [14]; and, finally, to exercise their clinical leadership, actively participating in the decision-making processes on the patient care pathway [25]. This method of team coordination and management promotes a culture of safety and quality of care [15] by removing barriers, leveraging facilitators, and making tangible changes in clinical care delivery [28,29].

The management of HAIs can be hindered or facilitated by several factors within the healthcare organization, including the work environment of the hospital or ward, the characteristics of the healthcare worker’s intervention, and, ultimately, the nature of the process [28]. Although the factors that influence implementation efforts are evident, the impact of the head nurse’s leadership on outcomes for patients with HAIs is often unclear.

**Figure 1:**
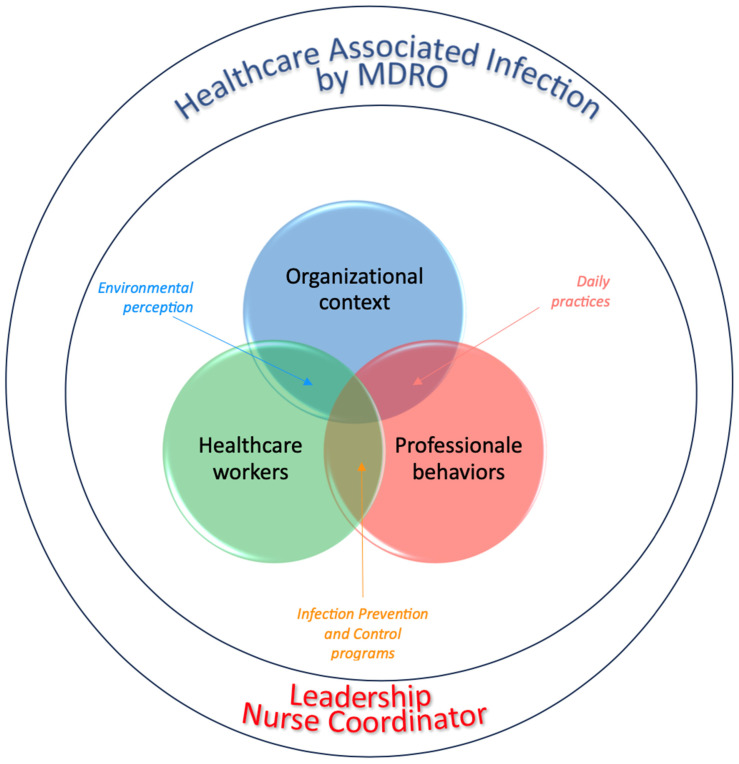
Theoretical framework.

Prior quantitative research has already identified leadership as a key organizational factor related to the safety and quality of care delivered [10,15,29]. The importance of the head nurse’s role in preventing and controlling HAIs was evaluated through studies comparing transformational and authentic leadership styles [24,25], organizational team well-being [19], and HAIs in care settings [15,25,26,30]. To date, no studies have examined how interventions from the head nurses could affect the clinical and organizational behaviors of the clinical care teams being managed. Examining the impact of the head nurse’s role in the prevention and control of HAIs through a qualitative approach can offer valuable insights into the effects of organizational and managerial decision-making on both the team and individuals. This approach could shed light on the potential intervention alternatives for healthcare facilities in the management of infection risks (Figure 1).

## 2. Materials and Methods

### 2.1. Objective

The purpose of this study is to examine how the behaviors and actions of the head nurse can promote compliance with Infection Prevention and Control (IPC) programs across the clinical care team.

To achieve this objective, we will:

- Describe the thought processes that motivate healthcare professionals in the care provided for reducing HAIs.
- Identify the barriers and facilitators to the implementation of IPC programs in clinical practice.

### 2.2. Design

The study will be a Grounded Theory [31,32] multi-center qualitative study. Non-participant observation will be followed by single interviews and/or focus groups. This approach enables the events to be observed as they occur, whereas the individual interviews provide a greater understanding of the thoughts behind each practitioner’s performance. The COnsolidated criteria for REporting Qualitative research (COREQ) checklist will be used for the reporting of this research [33] (see Supplementary Materials File).

### 2.3. Recruitment

The study will be conducted in at least two hospitals with equivalent IPC programs, according to WHO recommendations [5]. Healthcare facility managers from multiple hospitals will be invited to participate in the study, and, if enrolled, the respective epidemiological offices will be requested to complete the WHO Infection Prevention and Control Assessment Framework (IPCAF) [34]. During data analysis, we will compare participating healthcare facilities that have similar IPCAF scores.

### 2.4. Participants

The study will use a convenience sampling method, in which wards and related healthcare professionals who voluntarily choose to participate will be included. The study will include head nurses, nurses, nursing assistants, and clinicians who have worked in the selected ward for at least six months. The purpose is to understand the work organizational dynamics and leadership style of the head nurse and how it affects the thought processes of the clinical-care team because the theoretical framework sustains that the head nurse inspires and motivates healthcare professionals to collaborate and work together to ensure optimal patient care [13].

For the purposive sampling, a variety of settings will be ensured, including medical, surgical, and intensive care units. We will include healthcare professionals who are involved in direct patient care activities, both on and off shift, regardless of their employment contract (full-time/part-time, temporary/permanent). Healthcare professionals who do not give their consent to the study, those who work in the infectious diseases ward as they have IPC procedures dedicated to the isolation of specific pathogens, and managers will be excluded.

Since a qualitative method will be used, once the data have been saturated, participant enrollment will end [35]. A minimum of four professionals from each healthcare setting will be enrolled to collect and examine different clinical and work perspectives. These professionals will include head nurses, direct patient care nurses, nursing assistants, and clinicians.

### 2.5. Data Collection

After the individual wards have been identified and enrolled, an e-mail invitation to participate in the study will be sent to the head nurses, nurses, nursing assistants, and clinicians. A multidisciplinary group of participants will be selected. Participants will be informed about the aims of the project and asked to consent to being observed during their daily activities. They will then be interviewed regarding the activities observed. The number of care activities and processes performed during the day will determine the number of observations required. A minimum of ten observations for each hospital are expected to be conducted.

### 2.6. Instruments

To mitigate the Hawthorne effect and potential inter-observer biases [36], observations will be conducted by at least two experienced professionals who have previously participated in audits and infection risk management (e.g., hand hygiene audits). One observer will belong to the ward, and the other will be from the healthcare organization where the study is conducted. The observed activities will last approximately three hours and will be recorded in a logbook to reduce researcher subjectivity. Observations will focus on the behaviors and actions related to the following care activities or processes, according to international guidelines and scientific evidence [5,34,37].

1. The appropriate use of standard precautions in the management of HAIs caused by devices such as Central Line-Associated Bloodstream Infection (CLABSI) and Catheter-Associated Urinary Tract Infection (CAUTI).
2. The appropriate use of standard and additional precautions in the management of device HAIs caused by MDROs.
3. The use or non-use of cohorting staff in the case of patients with an HAI due to MDRO.
4. The management of in-hospital transport of a patient with an HAI caused by an MDRO.
5. The adherence of out-of-ward consultants to the proper use of standard and additional precautions in the case of a patient with HAI due to MDROs.
6. The management of environmental sanitation and the relations between healthcare personnel and out-of-hospital cleaners (e.g., patients’ cleaning order from non-infected to infected).

Following the observations, an expert will conduct individual interviews and/or focus groups to understand why the staff member behaved or acted as described. The interviews and focus groups will be recorded with a digital audio recorder, with prior explanation and informed consent from the person concerned. The researcher will also collect field notes. Each question is designed to allow the participant to describe how they perceive the topic of the study. The interview will be guided by open-ended questions with the dual purpose of creating a welcoming, non-judgmental environment [38] and understanding the thought processes that accompany the actions of the healthcare professionals (Table 1).

Finally, participants will be asked to provide their biographical information, including gender, age, qualifications, education, and work experience, and whether they have participated in training programs at their hospital on Infection Prevention and Control, management of HAI or infections caused by MDRO, or antibiotic stewardship.

### 2.7. Data Analysis

The data will be analyzed using a qualitative descriptive approach [39]. The interdisciplinary analysis will be conducted by an occupational psychologist, a qualitative research expert, and a university professor specializing in Grounded Theory. The observations will provide a general understanding of the environment, the participants, and the behaviors of professionals related to the management of HAIs. Instead, the interviews will be read multiple times and coded as appropriate. The interviews will be coded in the following three phases:

First phase: Open/initial coding, where transcripts will be read and parts of the text containing units of meaning related to the description of healthcare professionals’ thought processes that influence their behavior and actions will be selected. The importance of these units of meaning will depend on the research question(s). Additionally, the researcher’s field notes and observations will be included.

Second Phase: Final Coding—After open-coding all interviews, the texts and notes will be reread and the macro-categories will be identified, which provide a summary of the activities—both evident and unseen—that emerge from analyzing the interviews.

Third phase: Theoretical Coding—Among the macro-categories, the central categories will be identified, which must be significant and recurring, and possible connections between them will be suggested. Based on the results, a detailed description will be provided by presenting discussions that are appropriate for the research focus.

### 2.8. Methodical Rigor

The research team is composed of expert professors in the sector of nursing sciences who are experienced in clinical, organizational, qualitative, and occupational psychology research. This composition enables an understanding of the operational reality of nursing contexts, facilitating the observations and recruitment of participants for individual interviews and/or focus groups. During the construction, translation, and interpretation phases, the researchers, as instruments of inquiry, will strive to avoid potential biases or overestimations that could lead to theoretical “distortions” [38]. To manage and control subjectivity, reflexivity [31] and active confrontation within the team will be used to limit faulty or preconceived interpretations [40]. Researchers will explicitly clarify their backgrounds and experiences to participants to help them understand the context and dynamics present in the selected wards [41]. This awareness is fundamental to successful grounded research. Finally, direct observation by experienced healthcare professionals from the same ward can avoid behavioral assessment limitations.

### 2.9. Reliability

Following the COREQ criteria [33], the researchers will adhere to the basic steps of qualitative research and its theoretical interpretation. A detailed methodological description of the activities observed will be provided to facilitate comparison with the context, promote the replicability of the design, and enhance the reliability of the results. Finally, the study will be conducted by qualitative experts with extensive experience in the field to strengthen the theories developed.

### 2.10. Ethical Considerations and Dissemination

The principal investigator guarantees that the study will be conducted according to the protocol, Good Clinical Practice, the current version of the Declaration of Helsinki, and applicable regulations [42] and has been approved by the Ethics Committee of the Tor Vergata Hospital University–Rome with the following protocol number (Prot. N° RS 104.23).

The objectives of the study and the confidentiality of the data will be explained to all participants during a preliminary meeting. Participation in the protocol study is voluntary, and any healthcare professionals may refuse to participate at any time. Before partaking in any procedure related to this study, all participants must sign the informed consent.

## 3. Discussion

This study examines how head nurses’ behaviors and actions can promote clinical care team adherence to Infection Prevention and Control (IPC) programs. The decision to use a Grounded Theory approach is based on its intrinsic characteristics, such as its focus on the processes of meaning in research practice, language analysis, and the underlying processes that characterize social interaction [43]. Since care processes are characterized by a common constant, which is the interaction between different healthcare professionals within the work context, this research aims to define an interpretive theory [38] that will provide a better understanding of the factors responsible for the proper governance of HAIs.

Through the results of this study, we expect to outline the role of the head nurse within their team [44] and their capability to motivate them to ensure a performance that guarantees quality of care [15]. Additionally, it will analyze the challenges and requirements of the head nurse and their team in adopting IPC programs and managing HAIs from multi-resistant pathogens [14]. Targeted strategies can be developed from these considerations to enhance professional, technical, and non-technical competencies and skills for managing infectious risks in clinical practice.

The theoretical framework will be developed based on observations of care processes, patient interactions, clinical care procedures, and environmental sanitation activities. This question will be discussed, and understanding will be gained on how the figure of the head nurse could become a way to intervene in the ward and across the entire multidisciplinary team. The theoretical framework is essential for the prioritization of appropriate and innovative interventions that are responsive to the needs of head nurses and healthcare professionals. Describing the thought processes that motivate healthcare professionals in providing care to reduce HAIs will benefit healthcare managers by reducing the costs associated with negative patient outcomes.

Furthermore, these findings have great potential because they explore the perceptions of all healthcare professionals involved in the care process, care coordinators, nurses, nursing assistants, and clinicians. This approach considers potential barriers to using HAI programs and facilitators that could reduce the incidence of HAIs. These findings will be essential in informing policy decisions and improving day-to-day practice through the identification of potential internal changes. An understanding of the needs of head nurses and the clinical care team can provide insight into the actual unmet needs and the support necessary to implement new organizational and training strategies appropriate to the work context. Finally, identifying potential facilitators or barriers can ensure that all stakeholders can act intentionally and concretely. This will facilitate the development of synergistic collaborations with a focus on the significance of the infectious outcome in the care pathway of a hospitalized patient.

### Limitations

This study has some limitations due to the methods of data collection, observation activities, and interviews. The interpretation of operational reality will be compared to that of other researchers. To minimize subjectivity, a diary will be used. Additionally, interviewees’ perceptions and feelings may deviate from reality due to biases, such as a lack of trust in ensuring anonymity. To ensure confidentiality and anonymity, we will rely on the researchers’ experience in handling participants’ information, values, and beliefs.

## 4. Conclusions

The head nurse, with the support of the organization, is responsible for promoting a culture of safe care within the team. Field observations and interviews with key decision-makers in the team are needed to understand the impact of the head nurse’s leadership in the management of HAIs. Furthermore, analyzing the perceptions of head nurses, nurses, nurse assistants, and clinicians regarding the barriers and facilitators to adhering to HAI programs could be informative. Developing strategies to minimize the impact of barriers and focusing on factors that promote adherence to evidence-based best practices may help improve compliance with IPC programs and ensure quality care for patients with multi-drug-resistant HAIs.

## Figures and Tables

**Table 1:** Guidelines for conducting interviews.

Objectives	Main Questions	Supplementary Questions/Points of Attention
Background knowledge of the head nurse/nurse	Can you tell me about your education and work experience as a nurse and/or as a head nurse in the ward?	
Knowledge of the subject that is being studied	What do you think of when I say healthcare-associated infections/community infections/MDRO?	
Knowledge of the most important aspects of the prevention and control of HAI/HAI due to MDRO/community-acquired infections in clinical practice	Can you tell me about an event that occurred in your ward in the last month with a patient who was diagnosed with HAI/HAI from MDRO/community infection?	What did you think about this situation? What did you do at the time? How did you arrive at this solution? What were the alternatives to this solution? Can you describe to me the reaction of the patient/caregiver/relative to the diagnosis of HAI/HAI from MDRO/community infection? Do you think this could have been avoided?
Head Nurse’s Knowledge of Leadership Strategies for Managing and Controlling HAIs/HAs from MDROs/Communicable Infections	Can you tell me what strategies you use to increase your team’s adherence to hand hygiene/isolation of the patient with an HAI from an MDRO? Can you describe the key precautions and behaviors you use to manage a patient with HAI caused by MDRO?	Were these decisions shared with your staff or the staff of the IPC? Were they standard procedures or custom procedures? What was the role played by these professionals? Did you consider other hypotheses/interventions? Was this the first time you faced this situation?
Understand the thought processes that accompany and motivate contextualized decisions in your care setting	From your point of view, what are the strategies for communication and management of the problem of infection within your team?	(Contextualize the question about the organizational context and the patient’s clinical care needs.) How intense are these sharing and confrontation moments? Do you have an agreement with the physician or IPC nurses about the management of these issues? How do your staff approach these strategies?
Knowledge of barriers to IPC programs in clinical practice	From your perspective, what barriers and facilitators in your hospital might influence the implementation of IPC programs?	From your point of view, what could be changed in the implementation of IPC programs in your hospital? How do you think the relationship with specialist nurses of infectious risk could be defined? Give me an example of a clinical care process that most exposed patients to HAIs or MDRO HAIs. What strategies do you think your hospital uses to ensure safe and quality care for patients and staff?
Learning about health professionals’ experiences, expectations, and motivations	What was it like for you to participate in this interview? Do you have any questions?	

## Data Availability

No new data were created or analyzed in this study. Data sharing is not applicable to this article.

## Supplementary Materials

The following supporting information can be downloaded at: https://www.mdpi.com/article/10.3390/nursrep14030138/s1. Supplementary S1. COREQ (COnsolidated criteria for REporting Qualitative research) Checklist. Developed from: Tong et al., 2007 [33].

## Public Involvement Statement

There was no public involvement.

## Guidelines and Standards Statement

This manuscript was drafted against the COnsolidated criteria for REporting Qualitative research (COREQ) protocol.
