# Supplementary material for: Head Nurse Leadership: Facilitators and Barriers to Adherence to Infection Prevention and Control Programs—A Qualitative Study Protocol
Source: Nurs Rep. 2024 Jul 26;14(3):1849–58. doi: 10.3390/nursrep14030138 (PMC11348038; doi:10.3390/nursrep14030138)
Supplement: Supplementary file 1 [file nursrep-14-00138-s001.zip › nursrep-2982378-COREQ (COnsolidated criteria for REporting Qualitative research) Checklist.pdf]

Supplementary File S1: COREQ checklist

Consolidated criteria for reporting qualitative studies (COREQ): 32-item checklist

Developed from:

Tong A, Sainsbury P, Craig J. Consolidated criteria for reporting qualitative research (COREQ): a 32-item checklist for interviews and focus groups. International Journal for Quality in Health Care. 2007. Volume 19, Number 6: pp. 349 – 357

Item No	Guide Questions/Description	Reported on Page #
Domain 1: Research team and reflexivity		
Personal Characteristics		
1. Interviewer/ facilitator	Which author/s conducted the interview or focus group?	EC, JF
2. Credentials	What were the researcher's credentials? E.g., PhD, MD	MSN
3. Occupation	What was their occupation at the time of the study?	Student
4. Gender	Was the researcher male or female?	F, M
5. Experience and training	What experience or training did the researcher have?	Phd Student
Relationship with participants		
6. Relationship established	Was a relationship established prior to study commencement?	NO
7. Participant knowledge of the interviewer	What did the participants know about the researcher? e.g. personal goals, reasons for doing the research?	Research objective
8. Interviewer characteristics	What characteristics were reported about the interviewer/facilitator? e.g. Bias, assumptions, reasons and interests in the research topic	Interest in the research topic
Domain 2: study design		
Theoretical framework		
9. Methodological orientation and Theory	What methodological orientation was stated to underpin the study? e.g. grounded theory, discourse analysis, ethnography, phenomenology, content analysis	Grounded Theory
Participant selection		

Item No	Guide Questions/Description	Reported on Page #
10. Sampling	How were participants selected? e.g., purposive, convenience, consecutive, snowball	Theoretical
11. Method of approach	How were participants approached? e.g., face-to-face, telephone, mail, email	Face to face
12. Sample size	How many participants were in the study?	About 20
13. Non-participation Setting	How many people refused to participate or dropped out? Reasons?	None
14. Setting of data collection	Where was the data collected? e.g., home, clinic, workplace	Workplace
15. Presence of nonparticipants	Was anyone else present besides the participants and researchers?	No
16. Description of sample	What are the important characteristics of the sample? e.g. demographic data, date	Demographic data
Data collection		
17. Interview guide	Were questions, prompts, and guides provided by the authors? Was it pilot tested?	Questions
18. Repeat interviews	Were repeat interviews carried out? If yes, how many?	No
19. Audio/visual recording	Did the research use audio or visual recording to collect the data?	Audio recording
20. Field notes	Were field notes made during and/or after the interview or focus group?	Yes
21. Duration	What was the duration of the interviews or focus group?	30 minutes
22. Data saturation	Was data saturation discussed?	Yes
23. Transcripts returned	Were transcripts returned to participants for comment and/or correction?	No
Domain 3: analysis and findings		
Data analysis		
24. Number of data coders	How many data coders coded the data?	None
25. Description of the coding tree	Did the authors provide a description of the coding tree?	Yes
26. Derivation of themes	Were themes identified in advance or derived from the data?	Derived from the data
27. Software	What software, if applicable, was used to manage the data?	None

Item No	Guide Questions/Description	Reported on Page #
28. Participant checking	Did participants provide feedback on the findings?	No
Reporting		
29. Quotations presented	Were participant quotations presented to illustrate the themes/findings? Was each quotation identified? e.g., participant number	Yes
30. Data and findings consistent	Was there consistency between the data presented and the findings?	Yes
31. Clarity of major themes	Were major themes clearly presented in the findings?	Yes
32. Clarity of minor themes	Is there a description of diverse cases or a discussion of minor themes?	Yes
